# Absence of RstA results in delayed initiation of DNA replication in *Escherichia coli*

**DOI:** 10.1371/journal.pone.0200688

**Published:** 2018-07-16

**Authors:** Yuan Yao, Yong Ma, Xiuli Chen, Rengui Bade, Cuilan Lv, Runxiu Zhu

**Affiliations:** 1 Department of Neurology, Inner Mongolia People's Hospital, Hohhot, Inner Mongolia, China; 2 School of Life Sciences, Inner Mongolia University, Hohhot, Inner Mongolia, China; 3 Department of Biological Sciences and Technology, Baotou Teacher's College, Baotou, Inner Mongolia, China; 4 The Key Laboratory of Hypoxic Translational Medical, Baotou Medical College, Baotou, Innner Mongolia, China; Indian Institute of Science, INDIA

## Abstract

RstB/RstA is an uncharacterized *Escherichia coli* two-component system, the regulatory effects of which on the *E*. *coli* cell cycle remain unclear. We found that the doubling time and average number of replication origins per cell in an Δ*rstB* mutant were the same as the wild-type, and the average number of replication origins in an Δ*rstA* mutant was 18.2% lower than in wild-type cells. The doubling times were 34 min, 35 min, and 40 min for the wild-type, Δ*rstB*, and Δ*rstA* strains, respectively. Ectopic expression of RstA from plasmid pACYC-*rstA* partly reversed the Δ*rstA* mutant phenotypes. The amount of initiator protein DnaA per cell was reduced by 40% in the Δ*rstA* mutant compared with the wild-type, but the concentration of DnaA did not change as the total amount of cellular protein was also reduced in these cells. Deletion or overproduction of RstA does not change the temperature sensitivity of *dnaA46*, *dnaB252* and *dnaC2*. The expression of *hupA* was decreased by 0.53-fold in Δ*rstA*. RstA interacted with Topoisomerase I weakly *in vivo* and increased its activity of relaxing the negative supercoiled plasmid. Our data suggest that deletion of RstA leads to delayed initiation of DNA replication, and RstA may affect initiation of replication by controlling expression of *dnaA* or *hupA*. Furthermore, the delayed initiation may by caused by the decreased activity of topoisomerase I in RstA mutant.

## Introduction

Two-component systems are major signal transduction mechanisms in bacteria that are used to sense and respond to a huge variety of environmental stimuli. The two-component systems including a histidine kinase and a response regulator. This histidine kinase is autophosphorylated, and then the phosphoryl group is subsequently transferred to the response regulator at an aspartate residue.

RstB/RstA composes a two-component system in bacteria in which the histidine kinase, RstB, senses an extracellular signal, that autophosphorylates on a conserved histidine residue. RstB can transphosphorylate the response regulator, RstA, which have a conserved aspartate in receiver domain. Phosphorylated RstA can activate or repress target genes, thereby initiating a response to the extracellular stimulus. Previous studies have shown that response regulators TorR and BaeR can affect the DNA replication [1, 2]. However, until now, it has not been known how the RstB/RstA system affects the DNA replication. In the current study, we determined that the deletion of RstA delayed the initiation of DNA replication in *E*. *coli* cells.

## Materials and methods

### Bacterial strains, plasmids, and growth conditions

*E*. *coli* K-12 served as a wild-type cells. The strains and plasmids are listed in Table 1. The primers used were listed in Table 2. The *rstA* gene, along with its native promoter, was amplified from the genomic DNA of *E*. *coli* strain BW25113 [3] using primers rstA177-F and rstA177-R. DNA sequences of cloned fragments were confirmed to be correct by sequencing. The resultant fragment was inserted into the *Xho*I and *Hin*dIII sites of pACYC177 [4], generating plasmid pACYC-*rstA*. The promoter regions of *hupA* or *hupB* gene was amplified from the genomic DNA of *E*. *coli* strain BW25113 using primers hupAp-F/R or hupBp-F/R. The resultant fragment was inserted into the *Bam*HI and *Hin*dIII sites of pTAC3953 [5], generating plasmid p*hupA*p or p*hupB*p. The plasmid pACYC-*rstA*, p*hupA*p or p*hupB*p was transformed into cells using a method of CaCl_2_. For bacterial two hyper plasmids, the *topA* or *rstA* ORF without its terminal cordon was inserted into the pKNT25 vector or pUT18, respectively.

**Table 1:** Bacterial strains and plasmids.

Strains and plasmids	Genotype	Reference or source
BW25113	Wild type *rrnB3* ∆*lacZ4787 hsdR514* ∆(*araBAD*)*567*∆(*rhaBAD*)*568 rph-1*	[3]
MOR309	BW25113 *rstA*::*kan*	[3]
MOR457	BW25113 *rstB*::*kan*	[3]
YY6	MOR309/pACYC-*rstA*	This work
YY7	BW25113/pACYC-*rstA*	This work
YY8	MOR309/pACYC177	This work
YY10	BW25113/pACYC177	This work
MOR687	*dnaA46(Ts)*	This work
YY42	*dnaA46(Ts) rstA*::*kan*	This work
YY43	*dnaA46(Ts)*/pACYC-*rstA*	This work
MOR227	*dnaB252(Ts)*	This work
YY44	*dnaB252(Ts) rstA*::*kan*	This work
YY45	*dnaB252(Ts)*/pACYC-*rstA*	This work
MOR166	*dnaC2(Ts)*	This work
YY46	*dnaC2(Ts) rstA*::*kan*	This work
YY47	*dnaC2(Ts)*/pACYC-*rstA*	This work
pACYC177	rep_p15A_Ap^R^ (*bla*) Km^R^	[4]
pACYC-*rstA*	*rstA* gene on pACYC177	This work
pTAC3953	rep_pMB1_Ap^R^lacZ	[5]
p*hupA*p	*hupA* promoter fused to the *lacZ* gene on pTAC3953 (pTAC3953 derivative)	This work
p*hupB*p	*hupB* promoter fused to the *lacZ* gene on pTAC3953 (pTAC3953 derivative)	This work
BTH101	F *cya-99 araD139 galE15 galK16 rpsL1* (Str^r^) *hsdR2 mcrA1 mcrB1*	[7]
pKNT25	rep_p15A_Km^R^ placT25(pSU40 derivative)	[8]
pUT18	rep_ColE1_Ap^R^ placT18(pUC19 derivative)	[8]
pKNT-*topA*	*topA* fused to T25 on pKNT25	This work
pUT*-rstA*	*rstA* fused to T18 on pUT18	This work
pCA24N	rep _pMB1_Cm^R^*lacI*^*q*^p_pT5-*lac*_t_*his*_GFP	[9]
pRstA-GFP	*rstA* gene on pCA24N (pCA24N derivative)	[9]
BL21-Gold (DE3)	*E*. *coli* B F^-^*ompT hsdS*B (r_B_^-^m_B_^-^) *dcm*^*+*^ Tet^r^ *gal* (DE3) *endA* Hte	Agilent Technologies
YY97	BL21(DE3)/pRstA-GFP	This work

**Table 2:** Primers used.

Name of primer	Sequence	Purpose
hupAp	F5’-CGGGATCCCGTGATTTAACGCCTGATTTG	For inserting the promoter region of *hupA* into pTAC3953
	R5’-CCAAGCTTGTTATCCTTACAATGTGTTTATC	
hupBp	F5’-CGGGATCCGCGCTGCAAAATGAACCGTC	For inserting the promoter region of *hupB* into pTAC3953
	R5’-CCAAGCTTCCTCTTTATAATTTATATCGCAC	
rstA177	F5’-CCCTCGAGCCAGTTGCTTTGTCACCGGAC	For inserting *rstA* into pACYC177
	R5’-CCAAGCTTCGCTTATTCCCATGCATGAGG	
himA-Q	F5’-AACGGCGAACAGGTGAAACT R5’-GGGAATATCCTCGCCCGTTT	For RT-qPCR.
hip-Q	F5’- GGAGCATATGGCCTCGACTC	For RT-qPCR.
	R5’- ATTACGTCCGGTACGTGGTG	
hupA-Q	F5’- CCTTCAAAGTGAACCACCGC	For RT-qPCR.
	R5’- CCTTCAAAGTGAACCACCGC	
hupB-Q	F5’- GCCGTTAAAGAGCGTGCTG	For RT-qPCR.
	R5’- TTACCTGCACGGAAGCTCG	
rplO-Q	F5’- ATTCGGCTTCACTTCTCGTAA	For RT-qPCR.
	R5’-CTTTCAGCGTGTTCAGGTCTA	
topA-H	F5’- GCTCTAGAATGGGTAAAGCTCTTGTCATC	For inserting *topA* into pKNT25
	R5’- GGGGTACCCTTTTTTTCCTTCAACCCATTTG	
rstA-H	F5’-AACTGCAGATGAACACTATCGTATTTGTGGAAG R5’-CGGGATCCTTCCCATGCATGAGGCG	For inserting *rstA* into pUT18

*E*. *coli* cultures were cultured to OD_450_ = 0.15(optical density at 450 nm) in ABTGcasa medium [6] at 37°C. Ampicillin (50 μg/ml), chloramphenicol (15 μg/ml), tetracycline (50 μg/ml) and kanamycin (50 μg/ml) were added when required for selection.

### Flow cytometry

Cells were grown to OD_450_ = 0.15 in ABTGcasa medium, and then supplemented with rifampicin (300 μg/ml) and cephalexin (10 μg/ml) at 37°C for three to four generations. Adding the rifampicin in the cultures resulted in the inhibition of transcription, but rifampicin still allowed to complete the in-progress replication. The dosage of cephalexin prevents the cell division [10, 11]. The cells were fixed in 70% ethanol and then washed in Tris-HCl buffer (pH 7.5). Immediately, the Hoechst 33258 was used to stain the cells, and then analyzed by flow cytometer (BD Biosciences, USA). A total of 10,000 cells were included for each analysis. The methods of preparing standard samples and analysis methods were described as mentioned earlier [12]. https://dx.doi.org/10.17504/protocols.io.p9ndr5e.

### Determination of total protein per cell

Cells were grown to OD_450_ = 0.3 in ABTGcasa medium at 37°C and then placed on ice. The cell culture was harvested by centrifugation at 13000 rpm for 10 minutes at 4°C. The harvested cells were washed in 1 ml of Tris-EDTA buffer, resuspended in 250 μL of Tris-EDTA buffer containing SDS (1%) and glycerol, and then boiled for 6 min [1]. We did a colorimetric assay to determine the total amount of protein in the fixed volume of cell extract (9 ml) (BCA kit, Pierce Chemical, Rockford, IL, USA) as described previously [8]. The number of cells in the initial volume of culture and the cellular protein levels were determined as described previously [13].

### Western blotting

The DnaA concentration of cell extract was determined by Western blotting as described previously [14]. The cell extracts were fixed, subjected to SDS-PAGE, and transferred to a polywinylidene difluoride (PVDF) membrane by semi-dry blotting [10]. The anti-rabbit antibody for DnaA was used as probed. The secondary antibody was also anti-rabbit IgG conjugated with horseradish peroxidase (HRP) (Abcam, UK). http://dx.doi.org/10.17504/protocols.io.qaddsa6.

### Total RNA extraction

Total RNAs of BW25113 strain and its derivative cells were isolated using Trizol reagent kit (TRIzol™ Plus, Invitroge, USA), following the manufacturer's instructions. RNA integrity was verified by electrophoresis on a 1.2% agarose gel containing formaldehyde, and post stained with 1.0 μg/ml ethidium bromide. The 23S/16S ratios of all samples measured using an AgilentBio analyser were found to be about 2:1. RNA purity was determined using the NanoDrop 2000C spectrophotometer (NanoDrop Technologies, Thermo Scientific™, USA) by finding the A260/A230 and A260/A280 ratios. The A260/A280 ratios of all samples were 1.9–2.1 and the A260/A230 ratios were 2.0–2.1, as described previously [2]. Both integrity and purity of the RNA samples met the requirements for the reverse transcriptional quantitative PCR (RT-qPCR) analysis [15]. http://dx.doi.org/10.17504/protocols.io.qafdsbn.

### Relative quantitative real-time PCR

The RT-qPCR assay was performed in a LightCycter 480 II Real-Time PCR System (Roche, Switzerland) using SYBR^®^*Premix ExTaq*^TM^II kit (TliRNaseH Plus) (TaKaRa, Japan). After an initial denature at 95 °C for 30 s, 40 cycles of 95°C for 10 s, 60 °C for 20 s were used in the assay. Then melting curves were performed immediately as described previously. The value of Ct from each reaction could automatically be given. Using the concentrations of template (the X axis) and threshold cycles (the Y axis), the relative standard curve was obtained. The amplification efficiency (E) was then calculated by the equation 1, and the gene dosage was determined using the equation 2 as described previously [16]. The Ct value for each reaction was automatically obtained, and the relative expression of target gene was calculated using the formula 2(^-ΔΔCt^). Expression values were normalized to that of the *rplO* gene as a reference. The experiments were repeated three times with three technical replicates for each experiment.

### β-galactosidase activity assay

Exponentially growing cells (1 ml) at 37°C in ABTGcasa medium were collected at OD_450_ = 0.1, 0.2, 0.3, 0.4 and 0.5, then mixed with cold toluene (0.1 ml) and kept on ice immediately. For measurement of β-galactosidase activity, 0.2 ml toluene-treated sample was added to 1 ml Z buffer (40 mM NaH_2_PO_4_, 60 mM Na_2_HPO_4_, 10 mM KCl, 1 mM MgSO_4_ and 50 mM β-mercaptoethanol, pH 7.0) containing 0.66 mg/ml o-nitrophenyl-β-D-galactopyranoside. The reaction was performed at 30°C until the color changed to yellow and stopped by addition of 0.5 ml 1 M Na_2_CO_3_, and the absorbance at OD_420_ was measured. The β-galactosidase activity was calculated by 1000×OD_420_/reaction time (min) ×OD_450_×0.2 ml [17]. http://dx.doi.org/10.17504/protocols.io.qabdsan.

### Bacterial two hybrid analysis

Plasmids and strain used in the bacterial two hybrid system (BCATH) are listed in Table 1. When two proteins interact, the T18 and T25 fragments can be combined together to catalyze the formation of cAMP. The synthesized cAMP activates the expression of the *lacZ* reporter gene, forming the blue colonies on plates containing X-gal and IPTG, whereas two proteins that do not interact will form white colonies. The BTH101 cells with a pair of plasmids expressing the proteins tested for interaction were cultured as previously described [18]. http://dx.doi.org/10.17504/protocols.io.qaedsbe.

### Expression and purification of protein

*E*. *coli* cells carrying the RstA expression plasmid (pRstA-GFP) were grown in LB medium with chloromycetin (100 mg/ml), and expression was induced by adding IPTG (final concentration of 1.0 mM). After harvesting by centrifugation, the cells were resuspended in 12 ml Lysis/Equilibration buffer including 120 μl lysozyme (10 mg/ml), and placed on ice for 30 min, and then extracted by sonication method. The supernatant was clarified by centrifugation, and incubated with His-Select Ni-NTA Agarose (Thermo Fisher Scientific, USA) over night at 4°C. The next day, the supernatant was loaded onto Ni-NTA affinity resin (Qigen, Germany). Proteins were examined for purity by SDS-PAGE and fractions containing pure protein were pooled and dialyzed. The extraction of proteins was analyzed by BCA protein concentration quantitative method. http://dx.doi.org/10.17504/protocols.io.qaadsae.

### Assay of topoisomerase I activity

Add 2 μl of 10×topoisomerase I reaction buffer and 400 ng pUC19 plasmid DNA (Takara, Japan) to each of a series of 1.5-ml microcentrifuge tubes on ice. Adjust volumes with distilled water so that the final reaction volume in each tube, including that of the protein or extract added in step 2, is 20 μl. Add various amounts of purified RstA or one unit *E*. *coli* topoisomerase I protein (NEB, USA) to the tubes, then incubate 10 min at 37°C. Add 4 μl of 6×loading dye to each tube and load contents on an 0.8% agarose gel. Run gel 2h at 5 to 10 V/cm. Stain gel with ethidium bromide, destain briefly with water, and photograph the gel illuminated with a UV transilluminator [19]. http://dx.doi.org/10.17504/protocols.io.p99dr96.

## Results

### Deletion of *rstA* results in delayed initiation of DNA replication

**Fig 1:**
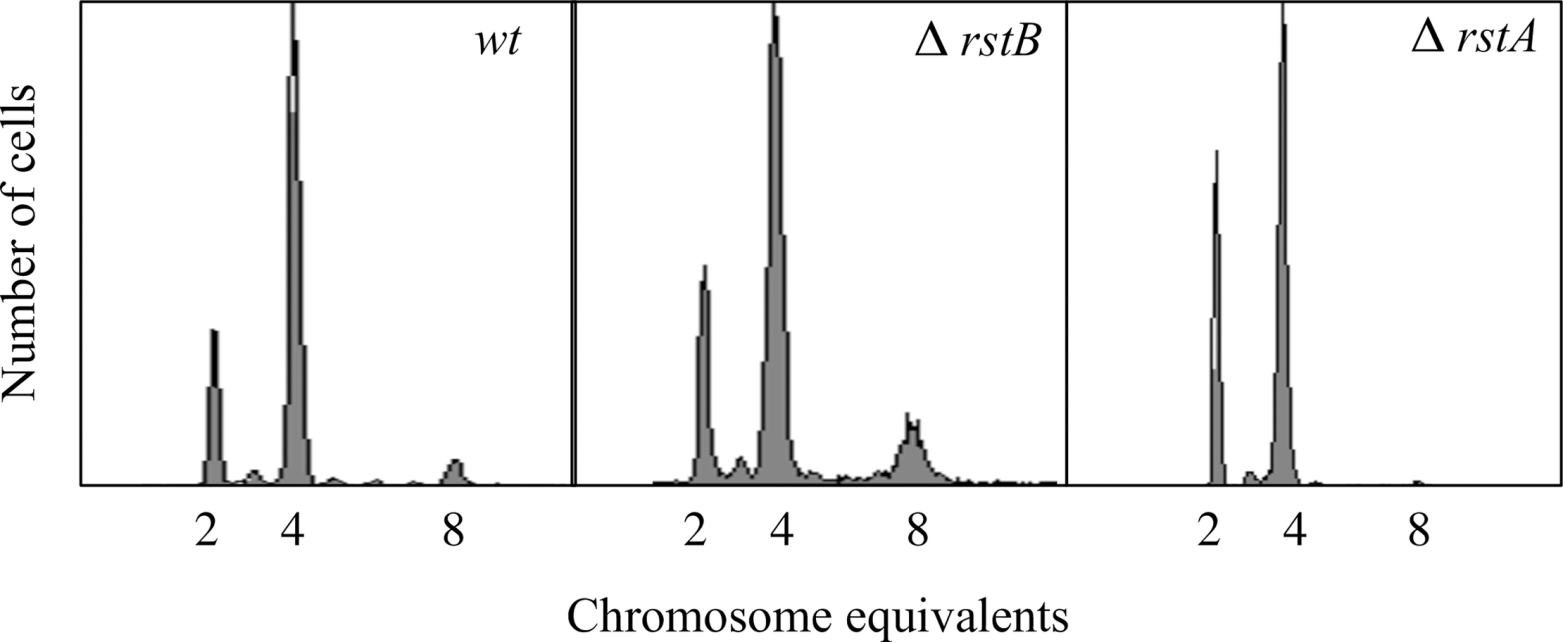
Deletion of *rstA* results in delayed initiation of replication. Cultures were grown to OD_450_ = 0.15 in ABTGcasa medium at 37°C, and then treated with rifampicin and cephalexin. After, 70% ethanol was used to fix the cells. The phenotype was detected by flow cytometry. The number of fully replicated chromosomes per cell represents the number of origins of replication present at the time of antibiotic addition. A total of 10,000 cells were used for each analysis.

We used the flow cytometry method to analyze the replication patterns of wild-type, Δ*rstB*, and Δ*rstA* cells for investigating the influence of RstB/RstA two-component system on the initiation of DNA replication. The cell cycle parameters were also compared among the Δ*rstB*, Δ*rstA* and wild-type cells (Fig 1, Table 3). The Δ*rstB* mutant had fewer cells in the B period and more cells in the D period compared with the wild-type cells. In the Δ*rstA* mutant, 24% of cells were in the B period, 75% were in the C period, and 1% were in the D period. Whereas the wild type had 20% of cells in the B period, 58% in the C period, and 22% in the D period. In addition, compared with the wild-type cells, Δ*rstB* mutant had the same proportion of 2-, 4- and 8-origin of replication cells, but Δ*rstA* mutant only contained 2- and 4- origin cells (Fig 1). The average number of origins of replication per cell was 4.4 for the wild-type cells, 4.3 for the Δ*rstB* mutant, and 3.6 for the Δ*rstA* mutant (Table 3). Concomitantly, the growth rate of the Δ*rstA* mutant also only little decreased, with doubling times of 34 min, 35 min, and 40 min recorded for the wild-type, Δ*rstB*, and Δ*rstA* cells, respectively (Table 3). The results suggested that Δ*rstA* mutants delayed the initiation of DNA replication compared to wild-type cells.

**Table 3:** Absence of RstA leads to a decrease in the number of origins per cell.

Strain	Cell cycle distribution (%)			A.O.	Doubling time (min)
	B-period	C-period	D-period		
BW25113	20±1.5	58±1.5	22±1.0	4.4±0.2	34±2
MOR457	11±1.0	60±1.5	29±1.5	4.3±0.1	35±3
MOR309	24±1.0	75±1.0	1±0.5	3.6±0.3	40±2
YY6	28±1.5	67±1.5	5±0.5	4.0±0.1	37±2
YY7	2±1.0	58±1.5	40±1.0	5.0±0.2	32±1
YY8	29±1.5	70±1.0	1±0.5	3.5±0.2	41±2
YY10	11±1.0	60±1.0	29±1.5	4.5±0.1	34±2

Exponentially growing cells in ABTGcasa medium were treated with rifampicin and cephalexin, fixed in 70% ethanol, and then analyzed by flow cytometry, as described in the Material and Methods section. The average number of origins per cell (A.O.) and the number of cells in B-, C- and D-period were calculated using software provided by BD Biosciences. Each experiment was repeated three times and standard errors are given.

### Ectopically-expressed RstA partly restores the delayed replication of the Δ*rstA* mutant

**Fig 2:**
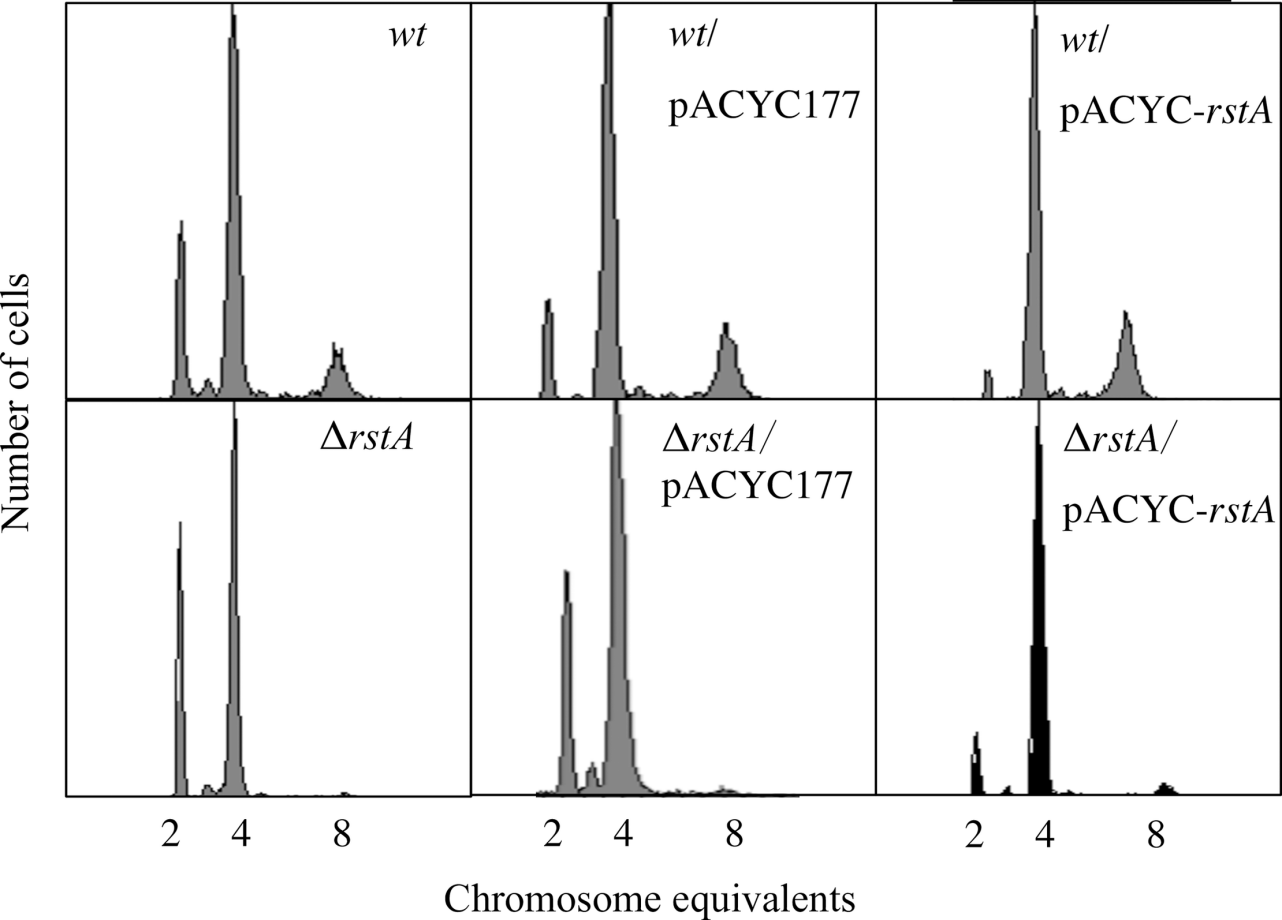
Ectopically-expressed RstA partially reverses the Δ*rstA* mutant phenotype. Cultures were grown to OD_450_ = 0.15 in ABTGcasa medium at 37°C to express RstA from the pACYC-*rstA* plasmid. Cells were treated and fixed as described in Fig 2. The chromosome number per cell was measured by flow cytometry.

Next, we wanted to determine whether RstA, which expressed by recombinant plasmids, could restore the delayed replication in the Δ*rstA* mutant. Over-expression of RstA in the Δ*rstA* mutant resulted in decreasing proportion of cells in C period (67% of cells) and increasing proportion of cells in the B (28%) and D (5%) periods compared with the Δ*rstA* strain (Table 3). In addition, the average number of origins of replication per cell increased from 3.6 in the Δ*rstA* mutant to 4.0 in the Δ*rstA*/ pACYC-*rstA* cells, and the doubling time decreased from 40 min in Δ*rstA* to 37 min in Δ*rstA*/pACYC-*rstA* cells (Fig 2, Table 3). When RstA protein was ectopically over-expressed in the wild-type cells (*wt*/pACYC-*rstA*), fewer cells were observed in the B period (2%), and more cells were detected in the D period (40%) compared with the wild-type cells (Fig 2). The average number of origins of replication per cell in *wt*/pACYC-*rstA* strain increased to 5.0 (Fig 2). Further, the doubling time of the over-expression strain decreased to 32 min (Fig 2, Table 3). Expression of the control plasmid in wild-type or Δ*rstA* cells did not affect replication. These results indicated that ectopically-expressed RstA could partially reverse the Δ*rstA* mutant phenotype. However, this *in trans* complementation would likely lead to other phenotypes, including bacterial filamentation and defects in septation.

### RstA affects the amount of DnaA per cell

**Fig 3:**
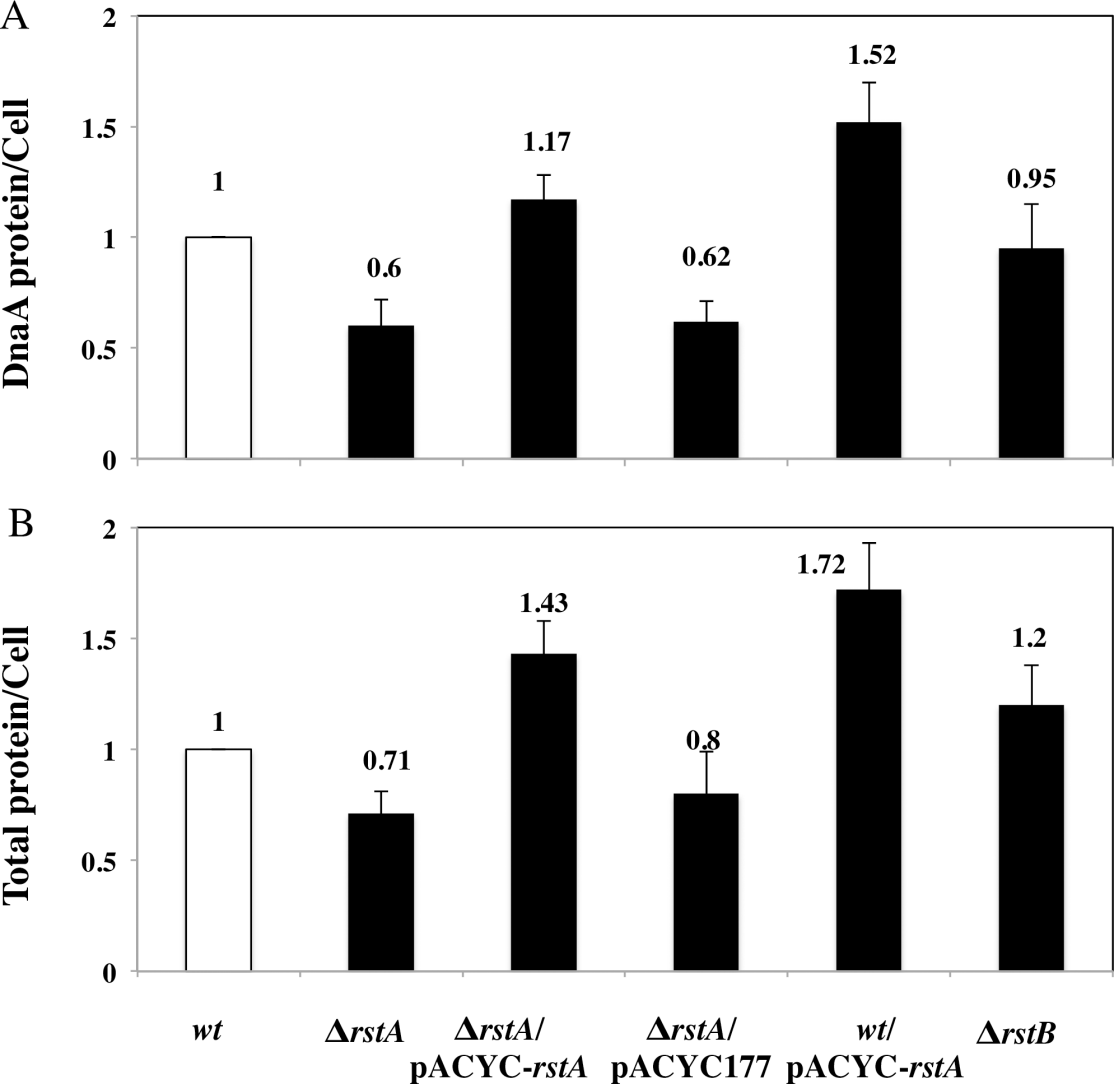
RstA affects the amount of DnaA per cell. **(**A) The amount of DnaA per cell was decreased in the Δ*rstA* mutant. Cultures were grown to OD_450_ = 0.3 in ABTGcasa at 37°C, and then harvested by centrifugation at 4°C. (B) The total amount of protein in a fixed volume of cell extract was determined by a colorimetric assay (BCA kit). The DnaA concentration was determined by immunoblotting. The amount of DnaA per cell was then estimated by counting the number of cells.

As a initiator of DNA replication, the amount and/or concentration of DnaA is a kind of limitation for the initiation of replication. To investigate whether RstA affects initiation of replication, we measured the amount of DnaA per cell in the wild-type, Δ*rstA* and Δ*rstB* cells. In addition, the western blotting was performed to measure the DnaA concentration in these cell extracts. The amount of DnaA per cell in the Δ*rstA* strain was reduced to 40% relative to the wild-type (Fig 3A). Further, we found that the total amount of protein per cell in the Δ*rstA* strain also decreased to 71% of that compared with the wild-type cells (Fig 3B). And the amount of DnaA per cell in the Δ*rstB* was similar to the wild-type (Fig 3B). The results indicated that the deletion of RstA delayed the initiation of replication by decreasing the total amount of protein (including DnaA) in the cell. RstA could regulate the expression of *dnaA*, and indirectly affect the DNA replication. In agreement with these findings, the expression of *dnaA* was decreased by 25% in ∆*rstB*∆*rstA E*. *coli* cells relative to the wild-type cells [20].

In conclusion, the deletion of *rstA* delayed the initiation of replication by decreasing the amount of DnaA and the total cellular protein levels in *E*. *coli*.

### RstA does not affect DNA replication through the functions of DnaA, DnaB and DnaC

The essential components for initiation of replication includes DnaA, DnaB and DnaC [9]. The mutant proteins DnaA46, DnaB252 and DnaC2 are thermo sensitive but functional at permissive temperature (30°C). However, these mutants could not survive at the non-permissive temperature (42°C) because of the blockage of replication [21]. So, we wanted to see whether RstA affected the initiation of replication by interacting with DnaA, DnaB or DnaC. The P1 transduction was used to introduce *rstA*::*kan*^*R*^ allele into the *dnaA46*, *dnaB252* and *dnaC2*, respectively [22]. And then the temperature sensitivity (*ts*) of above double mutants was determined. We found that these double mutants could not survive at the non-permissive temperature (42°C). Also, overproduction of RstA was not observed to change the survival rate of sensitive mutants (Table 4). These results suggested that deletion of RstA did not change the *ts* of the *dnaA46*, *dnaB252* and *dnaC2* mutants. Hence, it is possible that RstA does not affect DNA replication through the functions of DnaA, DnaB and DnaC.

**Table 4:** Deletion or overproduction of RstA does not change the temperature sensitivity of *dnaA46*, *dnaB252* and *dnaC2*.

Strain	Genotype	30°0	37°7	42°2
MOR687	*dnaA46(Ts)*	8/8	8/8	0/8
YY42	*dnaA46(Ts) rstA*::*kan*	8/8	8/8	0/8
YY42	*dnaA46(Ts)*/pACYC-*rstA*	8/8	8/8	0/8
MOR227	*dnaB252(Ts)*	8/8	8/8	0/8
YY44	*dnaB252(Ts) rstA*::*kan*	8/8	8/8	0/8
YY45	*dnaB252(Ts)*/pACYC-*rstA*	8/8	8/8	0/8
MOR166	*dnaC2(Ts)*	8/8	8/8	0/8
YY46	*dnaC2(Ts) rstA*::*kan*	8/8	8/8	0/8
YY47	*dnaC2(Ts)*/pACYC-*rstA*	8/8	4/8	0/8

The *rstA*::*kan*^R^ allele was transferred to *dnaA*46, *dnaB*252 and *dnaC*2 mutants by P1 transduction. The transductants were restreaked on LB agar plates with required antibiotics and then the survival ratio was tested by culturing the cells at 30° on LB agar p42°C.

### RstA regulates the expression of α-subunit of HU

**Fig 4:**
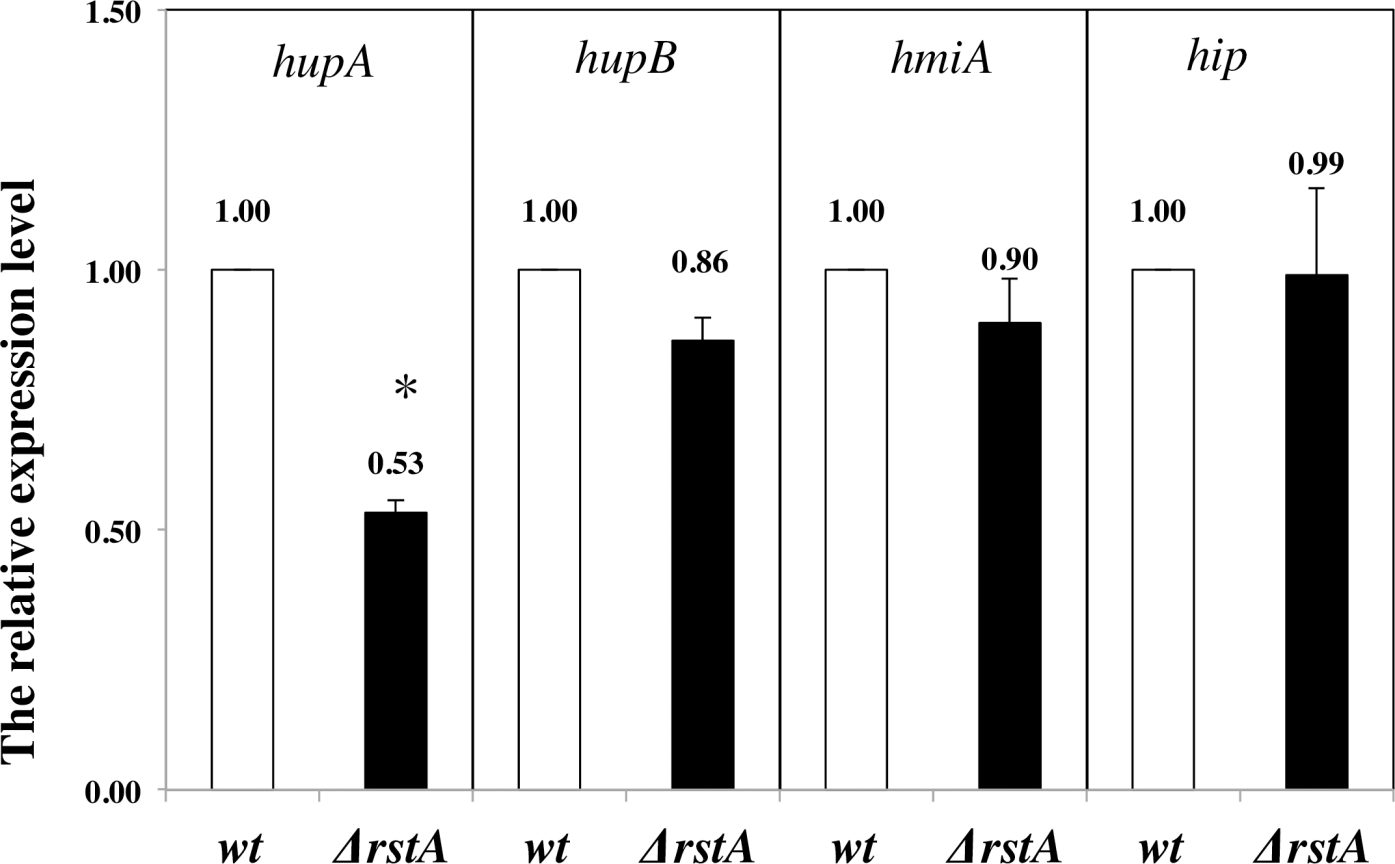
RstA regulates the expression of α-subunit of HU. The values shown at top of the bars are the average of three individual experiments, and the standard errors are shown. The significance of discrepancy between data by single factor analysis of variance. The ‘*’ showed significant differences between wild type and Δ*rstA* cells, *p*-value<0.001.

The homology proteins heat unstable protein (HU) and integration host factor (IHF) play a role in replication [23]. To find out possible reason behind this delay in initiation in Δ*rstA* strain, the relative expressions of IHF and HU were dertermined by RT-qPCR. The expression of *hupA* (α-subunit of HU) was 0.53 fold in Δ*rstA* compared with the wild type cells, but the expression of *hupB* (β-subunit of HU) was slightly decreaced (Fig 4). The IHF protein includes two subunits (HimA and Hip). However, there were no significant expressive difference of *himA* and *hip* between Δ*rstA* and the wild type cells (Fig 4). The above results indicated that RstA could regulate the expression of *hupA* to influence the replication.

**Fig 5:**
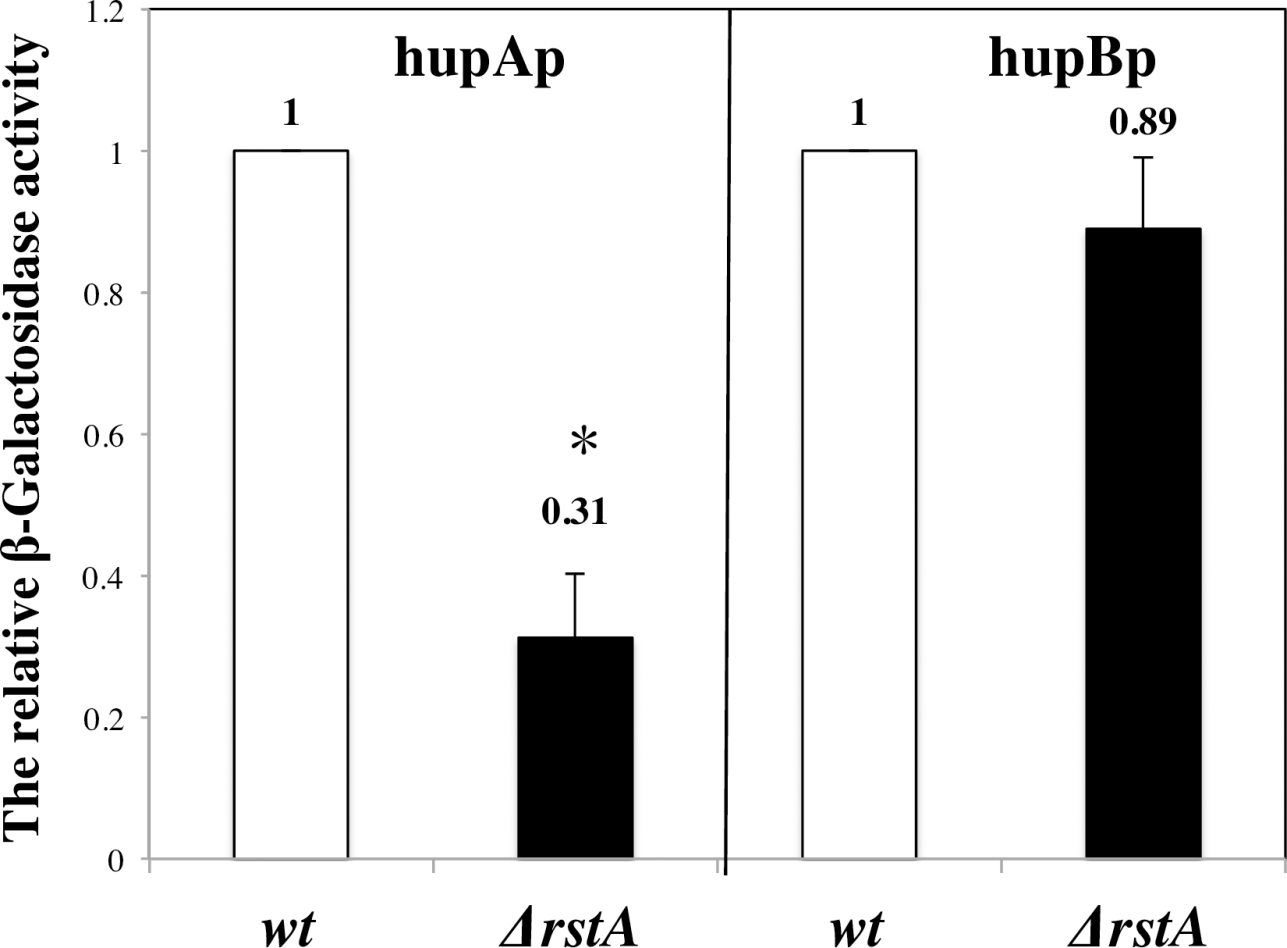
RstA interacts the promoter region of *hupA*. Cells were grown exponentially at 37°C in ABTGcasa medium and collected 1 ml at OD_450_ = 0.1, 0.2, 0.3, 0.4, 0.5, respectively. The sampled at the time points indicated and fixed in methylbenzene. Activity of the LacZ was measured as the β-galactosidase activity in the cells by Miller method. The values shown at top of the bars are the average of three individual experiments, and the standard errors are shown. The difference between data is analyzed by single factor analysis of variance. The ‘*’ showes significant differences between the wild type and Δ*rstA* cells, p-value<0.001.

To further explore the above hypothesis, the β-Galactosidase activity assay was performed. The promoter region of *hupA* or *hupB* was fused with *lacZ* gene in pTAC3953 plasmid. We detected the transcriptional activity of hupAp or hupBp in Δ*rstA* and the wild type cells respectively. Compared with the wild type cells, the hupAp activity decreased by 0.31 times in the Δ*rstA* mutant, and the hupBp activity was slightly decreased by 0.89 times without significant difference (Fig 5). The above results indicate that the activity of hupAp promoter is regulated by RstA.

### The RstA protein weakly interacts with topoisomerase I

**Fig 6:**
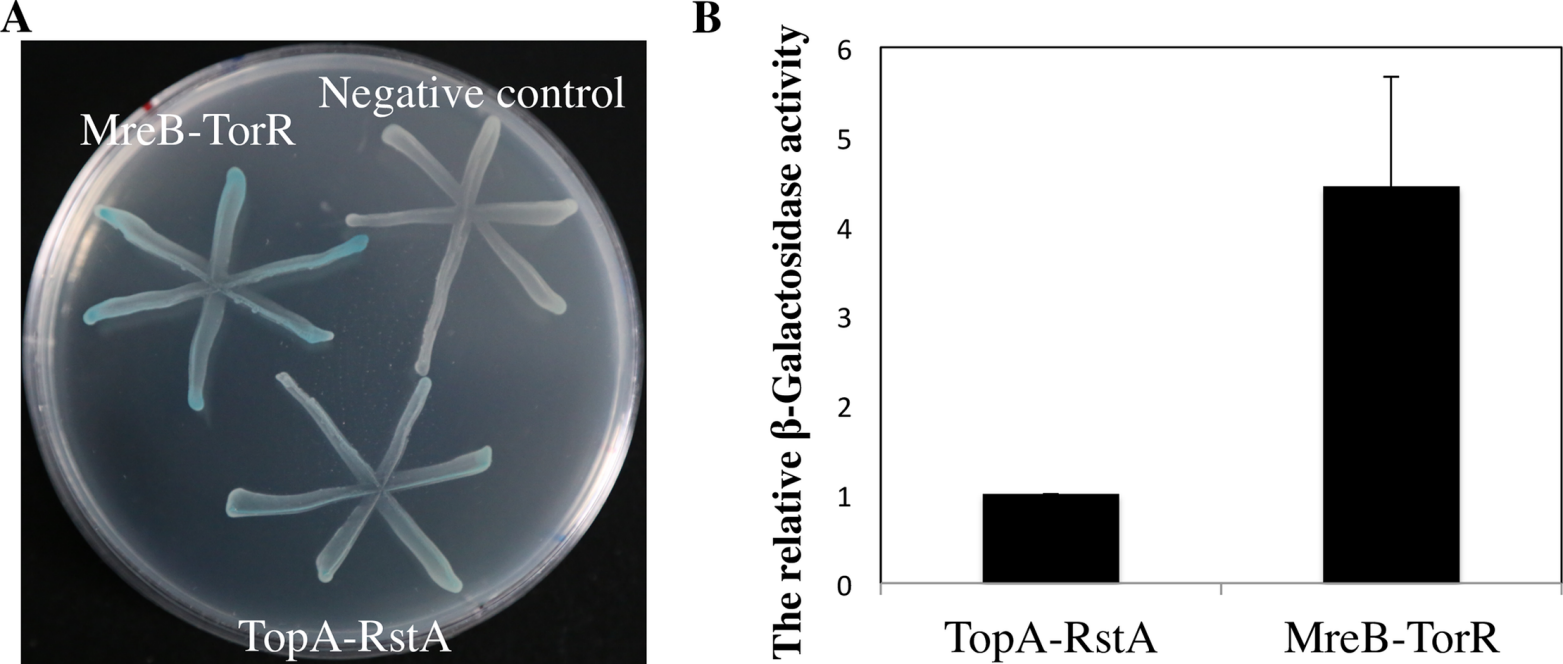
The TopA protein weakly interacts with RstA protein in *vivo*. (A) BTH101 cells were co-transformed with plasmid pairs encoding T18 and T25 respectively as negative control, then plated on the LB plates mentioned above with required antibiotics, incubated at 30°e for 30 hours. BTH101 cells were co-transformed with plasmid pairs encoding T18-TorR and T25-MreB respectively as positive control, proved previously. To exclude “false” blue colonies, 3 μl of the bacterial culture from each transformant after grown in LB for 2 hours was streaked in the same plate as described above, incubated at 30°t for 30 hours. All transformants illustrating different protein-protein interactions were simultaneously tested on one plate to have the same reaction condition. The blue bacterial streaks indicate protein-protein interactions while the white streaks show no interaction. Protein pairs under detection are as indicated. (B) The cells were treated and measured in β-galactosidase activity assay as described in Fig 5.

In *E*. *coli*, DNA topoisomerase I is encoded by the *topA* gene. Topoisomerase I is a type IA topoisomerase which is responsible for relaxing negative supercoils in DNA. Type I topoisomerases function by causing single-strand DNA breaks, while type II topoisomerases cause double-strand DNA breaks. So, we wanted to determine the protein-protein interaction of *E coli* that topoisomerase I interacts with RstA. To check the possibility, we detect the interaction of TopA with RstA *in vivo* by bacterial two hybrid system [7]. When two proteins physically interact in the bacterial two-hybrid system, the reporter *lacZ* gene can be expressed in a cAMP/CRP (cAMP receptor protein)-dependent way [7], producing blue colonies, otherwise white colonies, on LB plates containing X-gal. The cells expressing TopA and RstA resulted in very weak blue colonies whereas the positive control (TorR-MreB interaction) gave rise to blue colonies [2] and the negative control cell colonies remained white (Fig 6). The results indicate that TopA interacts with RstA weakly *in vivo* (Fig 6), suggesting that RstA might affect the initiation through this direct interaction between TopA and RstA.

### RstA increases the activity of topoisomerase I

**Fig 7:**
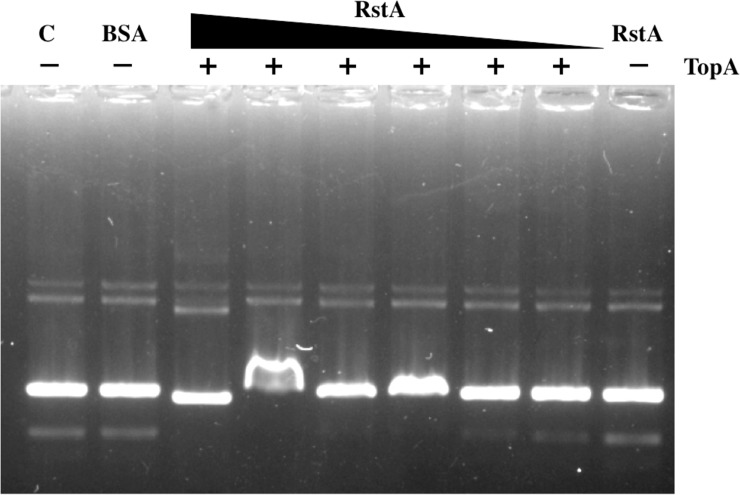
Topoisomerase I relaxes negative supercoiled plasmid DNA in present RstA. Lane 1, 0.4 μg pUC19 plasmid with no added protein. Lane 2, 0.4 μg pUC19 plasmid with BSA protein. And the other lanes contain the same concentrations of plasmid as lane 2, but different amounts of purified protein. Lanes 3–8 contain decreasing amounts of purified RstA protein (from 500 ng to 0 μg) and one unit Topoisomerase I. Lane 9, 500 ng purified RstA protein without Topoisomerase I. 6×loading buffer was added to stop the reaction and analyzed by 0.8% agrose gel electrophoresis.

We showed that the interaction of TopA-RstA previously. It could be possible that the RstA might affect the activity of TopA. To check this possibility, we firstly did the DNA relaxation experiment. A principal reaction of TopA is the relaxation of supercoiled DNA, which has a different electrophoretic mobility than DNA that is completely relaxed. Because plasmid DNA isolated from most natural sources is negatively supercoiled, pUC19 plasmid isolated from *E*. *coli* is used to assay topoisomerase I activity. As shown by the Topoisomerase I activity assay (Fig 7), RstA could not relax the supercoiled plasmid but enhance the activity of Topoisomerase I.

## Discussion

The DnaA protein plays an important role in initiation of replication in *E*. *coli*, which is subjected to regulation both regarding the nucleotide bound to the protein. The precise time of initiation of replication was controlled by the DnaA in *E*. *coli* [24]. We show here that the amount of DnaA per cell was decrease/increase in deleted/overproduced RstA strains, and the *ts* of *dnaA46*, *dnaB252* and *dnaC2* mutants was not changed (Fig 3, Table 4). The results suggest that the initiation of replication affected by RstA was not due to the variation of amount of DnaA per cell. In Δ*baeR* and *ftsA(ts)* mutants, the similar function of DnaA on DNA replication was also observed [1, 25].

The replication initiation protein DnaA interacts with the DnaA box on *oriC* to make DNA sequence bent. The HU protein modulates the binding of IHF to *oriC* and stabilizes the DnaA oligomer bound to *oriC* [26, 27]. With the help of DnaC, the DnaB is assembled in the form of six polymer in the replicating opening complex to form a pre priming complex. After that, the DnaG and DNA polymerase III are assembled into the pre primring complex and then the initiation of replication is triggered. We found that the expression of *hupA* was decreaseed in Δ*rstA* cells compared with the wild type cells. But the expression of *himA* and *hip* did not change between the wild type and Δ*rstA* cells. These results suggested the RstA might affect the initiation of replication through regulating the expression of α-subunit of HU. The Hu protein has been shown to be involved in DNA replication [28]. The lower amount of HU in Δ*rstA* cells might result the unstable combination between IHF and *oriC*.

Topoisomerase I relaxes negative torsional stress and is required to prevent the chromosomal DNA from becoming extensively negatively supercoiled [29]. Topoisomerase I suppresses initiation of replication at extraneous sites throughout the DNA duplex while permitting it to occur at the *oriC* sequence complexed by DnaA protein [30]. In this study, we employed a bacterial two hybrid assay to test TopA-RstA interaction, and the weak interaction was observed. Therefore, we believed that RstA might increase the activity of TopA through interacting with it and further affect initiation of replication. Subsequently, in the experiment of measuring the activity of topoisomerase I, the above hypothesis was proved. And the decreased activity of topoisomerase I in Δ*rstA* could be one possible reason for delay in initiation of replication. We also detected the relative expression of *topA* in Δ*rstA* cell, and found there was no change compared with the wild-type (The data was not shown), in accordance with the results of previous studies [20]. So, we believed RstA delayed the initiation of replication by means of changing the activity of TopA, but regulating the expression of TopA.
